# Melatonin Rescued Reactive Oxygen Species-Impaired Osteogenesis of Human Bone Marrow Mesenchymal Stem Cells in the Presence of Tumor Necrosis Factor-Alpha

**DOI:** 10.1155/2019/6403967

**Published:** 2019-09-05

**Authors:** Xianjian Qiu, Xudong Wang, Jincheng Qiu, Yuanxin Zhu, Tongzhou Liang, Bo Gao, Zizhao Wu, Chengjie Lian, Yan Peng, Anjing Liang, Peiqiang Su, Dongsheng Huang

**Affiliations:** ^1^Department of Orthopedics, Sun Yat-sen Memorial Hospital of Sun Yat-sen University, Guangzhou, Guangdong, China; ^2^Department of Orthopedics, The Third Affiliated Hospital of Sun Yat-sen University, Guangzhou, Guangdong, China; ^3^Department of Orthopedics, The First Affiliated Hospital of Sun Yat-sen University, Guangzhou, Guangdong, China; ^4^Guangdong Provincial Key Laboratory of Orthopedics and Traumatology, First Affiliated Hospital, Sun Yat-sen University, Guangzhou, Guangdong, China; ^5^Guangdong Province Center for Peripheral Nerve Tissue Engineering and Technology Research, Guangzhou, Guangdong, China; ^6^Guangdong Province Engineering Laboratory for Soft Tissue Biofabrication, Guangzhou, Guangdong, China

## Abstract

Accumulation of reactive oxygen species (ROS), which can be induced by inflammatory cytokines, such as tumor necrosis factor-alpha (TNF-*α*), can significantly inhibit the osteogenic differentiation of bone marrow mesenchymal stem cells (BMSCs). This process can contribute to the imbalance of bone remodeling, which ultimately leads to osteoporosis. Therefore, reducing the ROS generation during osteogenesis of BMSCs may be an effective way to reverse the impairment of osteogenesis. Melatonin (MLT) has been reported to act as an antioxidant during cell proliferation and differentiation, but its antioxidant effect and mechanism of action during osteogenesis of MSCs in the inflammatory microenvironment, especially in the presence of TNF-*α*, remain unknown and need further study. In our study, we demonstrate that melatonin can counteract the generation of ROS and the inhibitory osteogenesis of BMSCs induced by TNF-*α*, by upregulating the expression of antioxidases and downregulating the expression of oxidases. Meanwhile, MLT can inhibit the phosphorylation of p65 protein and block the degradation of I*κ*B*α* protein, thus decreasing the activity of the NF-*κ*B pathway. This study confirmed that melatonin can inhibit the generation of ROS during osteogenic differentiation of BMSCs and reverse the inhibition of osteogenic differentiation of BMSCs in vitro, suggesting that melatonin can antagonize TNF-*α*-induced ROS generation and promote the great effect of osteogenic differentiation of BMSCs. Accordingly, these findings provide more evidence that melatonin can be used as a candidate drug for the treatment of osteoporosis.

## 1. Background

Reactive oxygen species (ROS) are oxygen-containing chemically active substances. They include the hydroxyl radical (OH), hydroxyl ion (OH^−^) hydrogen peroxide (H_2_O_2_), and superoxide anion (O_2_^−^). ROS can be formed as a natural byproduct of cellular oxygen metabolism, mainly in the mitochondria. It also can be derived from nicotinamide adenine dinucleotide phosphate oxidase (NAPDH oxidase/NOX), peroxisome, xanthine oxidase, and lipolytic enzyme [1]. There are also antioxidant systems that can antagonize ROS production, such as superoxide dismutase (SOD), catalase (CAT), and glutathione (GSH), to maintain a normal level of ROS. Under normal physiological conditions, ROS remain at low levels with no obvious cytotoxicity and they can play an important role in cell signaling and homeostasis, promoting cell proliferation and differentiation [2]. However, stimulation of inflammatory cytokines such as tumor necrosis factor-alpha (TNF-*α*) can lead to large production of large amounts of ROS [3, 4]. Consequently, the high level of ROS can damage the normal functions of cells and even lead to cell apoptosis.

In the skeletal system, bone marrow mesenchymal stem cells (BMSCs) play an important role in bone formation. Generally, BMSCs can differentiate into osteoblasts, chondrocytes, and adipocytes [5]. Several studies have suggested ROS can affect the proliferation and differentiation of BMSCs. ROS at low levels are essential to BMSC signaling and homeostasis, while high levels of ROS lead to impaired functions and apoptosis of MSCs. Excessive ROS stimulation can regulate apoptosis-related gene expression by activating MAPK pathways such as the JNK, p38, and ERK pathways, leading to apoptosis in MSCs [6]. ROS can downregulate the osteogenic differentiation markers and cause osteogenic damage to the osteoblast cell line MC3T3-E1 [7]. Almeida et al. [8] found that increased production of ROS in mice can lead to decreased osteoblasts and bone formation. Moreover, ROS can also enhance adipogenesis of BMSCs and ultimately cause bone formation disorders through inhibition of Wnt/*β*-catenin and Hedgehog signaling [1]. Accordingly, it may be possible to counteract the inhibition of osteogenic differentiation of BMSCs by reducing ROS generation.

Melatonin (MLT) is a hormone produced mainly by the pineal gland. It has been shown to have a wide range of effect, including the inhibition of tumor growth, immunomodulation, and regulation of circadian rhythms [9]. In 1995, Reiter [10] proposed that melatonin can directly scavenge the free radicals. In addition, Antolin et al. [11] showed that melatonin can efficiently reduce the ROS generation by enhancing the activity of antioxidative enzymes. In recent years, Ganie et al. [12] also suggested that the function of melatonin to exert antioxidants is related to mitochondria. Many studies have reported that melatonin plays significant antioxidative roles in physiological and pathological processes, including the prevention of kidney and liver injury [13, 14] and protection of the spinal cord after ischemia [15]. Recently, more and more studies have been carried out to investigate the effect of melatonin during differentiation of MSCs. It has been demonstrated that melatonin can significantly enhance osteoblast differentiation of MSCs [16]. Similarly, our previous works have showed that melatonin is significant in promoting the osteogenesis and chondrogenic differentiation of BMSCs [17–21]. Among these relevant studies, most have focused on the development-promoting effect of melatonin during differentiation of BMSCs, whereas few of them have investigated its antioxidant effect in an inflammatory microenvironment, especially in the presence of TNF-*α*.

In this study, we investigated whether melatonin can decrease the ROS generation and thus protect osteogenic differentiation of BMSCs in an inflammatory microenvironment induced by TNF-*α*. The aims of this research are to further confirm the importance of melatonin as an antioxidant during the osteogenesis of BMSCs as well as to provide additional support for the use of melatonin as a candidate drug for the treatment of osteoporosis.

## 2. Material and Methods

### 2.1. Antibodies and Reagents

Antibodies against runt-related transcription factor-2 (RUNX2), osteopontin (OPN), NADPH oxidase1 (NOX1), and NADPH oxidase2 (NOX2) were purchased from Abcam (Cambridge, UK). Cu/Zn superoxide dismutase (Cu/Zn SOD or SOD1), catalase (CAT), NF-*κ*B signaling protein (p65, phosphor-p65, and I*κ*B*α*), goat anti-mouse IgG, and goat anti-rabbit IgG secondary antibodies were from Cell Signaling Technology Inc. (Boston, MA, USA). Abs against GAPDH was from Proteintech Group Inc. (Rosemont, IL, USA). Melatonin was purchased from Sigma-Aldrich (St. Louis, MO, USA), and recombinant human TNF-*α* was purchased from R&D Systems (Minneapolis, MN, USA). The osteogenic differentiation medium was purchased from Cyagen Biosciences Inc. (Santa Clara, CA, USA).

### 2.2. Isolation and Culture of Human Bone Marrow-Derived MSCs

The study was approved by the Ethical Committee of Sun Yat-sen University, and written informed consent was obtained from all participants. MSCs were isolated from bone marrow obtained from healthy volunteer donors (*n* = 3) as described previously [17]. Briefly, the bone marrow samples (8–10 mL) were diluted with 10 mL of phosphate-buffered saline (PBS). Cells were then fractionated on a Lymphoprep density gradient by centrifugation at 500 g for 20 min. Interfacial mononuclear cells were collected, resuspended in low-glucose Dulbecco's modified Eagle's medium (DMEM; Gibco, Waltham, MA, USA) supplemented with 10% FBS (Gibco), seeded, and incubated at 37°C/5% CO_2_. After 48 h, nonadherent cells were removed by changing the medium. Thereafter, the medium was changed every three days. When the cells reached 85%–95% confluence, they were trypsinized, counted, and plated again. Cells from passages 3–6 were used for the experiments.

### 2.3. Osteogenic Differentiation

BMSCs were cultured in a 6-well plate with complete medium. After reaching 80% confluence, the cells were cultured with osteogenic medium and treated with or without vehicle (0.05 mol/L ethanol, as used for melatonin dissolution), TNF-*α* (10 ng/mL), and melatonin (100 *μ*mol/L). The osteogenic medium was composed of low-glucose DMEM, dexamethasone (0.1 *μ*mol/L), 10% FBS, ascorbic acid (50 *μ*g/mL), and *β*-glycerol phosphate (10 mmol/L). The medium was changed every 72 hours.

### 2.4. Assessment and Measurement of ROS

ROS production in BMSCs was detected and measured by a fluorescence probe, 2′,7′-dichlorodihydrofluorescein diacetate (CM-H_2_DCFDA; Invitrogen, Waltham, MA, USA). Adherent cells in the 6-well plate were incubated with DMEM containing CM-H_2_DCFDA (10 *μ*mol/L) for 25 min at 37°C in darkness. Cells were washed with PBS three times, and fluorescence from the plate was photographed using the microscope (Leica DMI4000B; Leica, Wetzlar, HE, Germany). Suspension cells (2 × 10^5^; *n* = 3) were resuspended in DMEM containing CM-H_2_DCFDA (10 *μ*mol/L) and incubated for 25 min at 37°C in darkness. After washing with PBS three times, fluorescence intensity was quantified at 488 nm excitation and 525 nm emission by flow cytometry (CytoFLEX; Beckman Instruments Inc., Fullerton, CA, USA). Each sample contained 30,000 cells. Data was calculated by FlowJo (Becton, Dickinson and Company, Franklin Lakes, NJ, USA).

### 2.5. Alizarin Red S Staining

Cells were rinsed with PBS twice and fixed in formaldehyde (4%) for 10 min after 14 days of osteogenesis. After removing the fixing solution, the cells were stained with Alizarin red S (ARS) solution (Cyagen Biosciences Inc.) for 10 minutes at room temperature. After three washing with PBS, the cells were photographed. Calcium deposits are shown in red. Stains were measured at 405 nm to quantify the amount of Alizarin red. Each experiment was performed in triplicate.

### 2.6. Reverse Transcription Quantitative Polymerase Chain Reaction (RT-qPCR)

Total RNA was extracted from cells in different treatment groups using a RNAiso Plus reagent (TaKaRa, Dalian, China) and then converted to cDNA using PrimeScript RT Master Mix (TaKaRa) according to the manufacturer's protocols. qPCR was performed on a Light Cycler 480 Real-Time PCR Detection System (Roche, Basel, Kanton Basel-Stadt, Switzerland) using SYBR Green I Master Mix (Roche). Expression levels of the following genes were analyzed: *RUNX2*, *OPN*, *NOX1*, *NOX2*, *SOD1*, and *CAT.* The expression level of the glyceraldehyde-3-phosphate dehydrogenase (GAPDH) gene served as a reference. The Ct value of the GAPDH was subtracted from the Ct value of the target gene (ΔCt), and the average ΔCt value of the triplicates was recorded. The relative expression levels of each gene were determined using the 2-ΔΔCt method. Primer sequences used in this study are listed in Table 1.

### 2.7. Western Blot Analysis

Cells were rinsed twice with ice-cold PBS and lysed in RIPA lysis buffer (Beyotime, Shanghai, China) containing protease inhibitor cocktail (BioTool, Houston, TX, USA). Cell lysates were centrifuged at 12,000 g for 15 min at 4°C, and the protein concentration was quantified using the BCA protein assay kit (Beyotime). Equal amounts of each sample were subjected in 10% SDS-polyacrylamide gel electrophoresis (PAGE) and then transferred to PVDF transfer membranes (Millipore, Billerica, MA, USA). Membranes were blocked with 5% nonfat dry milk for 1 h at 25°C and then incubated with the designated antibodies at 4°C overnight. Antibody-specific labeling was observed by incubation with horseradish peroxidase- (HRP-) conjugated secondary antibodies for 1 h at room temperature, and the results were visualized with the ECL kit (Millipore). The bands were quantified by ImageJ software (National Institutes of Health, Bethesda, MD, USA) and normalized to GAPDH as the loading control.

### 2.8. Statistical Analysis

All quantitative data were given as mean ± SD. Statistical analysis was performed using one-way ANOVA followed by Dunnett's post hoc test for multiple comparisons. All statistical analyses were conducted with the SPSS 20.0 statistical software package (SPSS Inc., Chicago, IL, USA). *P* < 0.05 was considered statistically significant.

## 3. Results

### 3.1. Melatonin Reduced TNF-*α*-Induced ROS Generation during Osteogenesis of BMSCs

**Figure 1:**
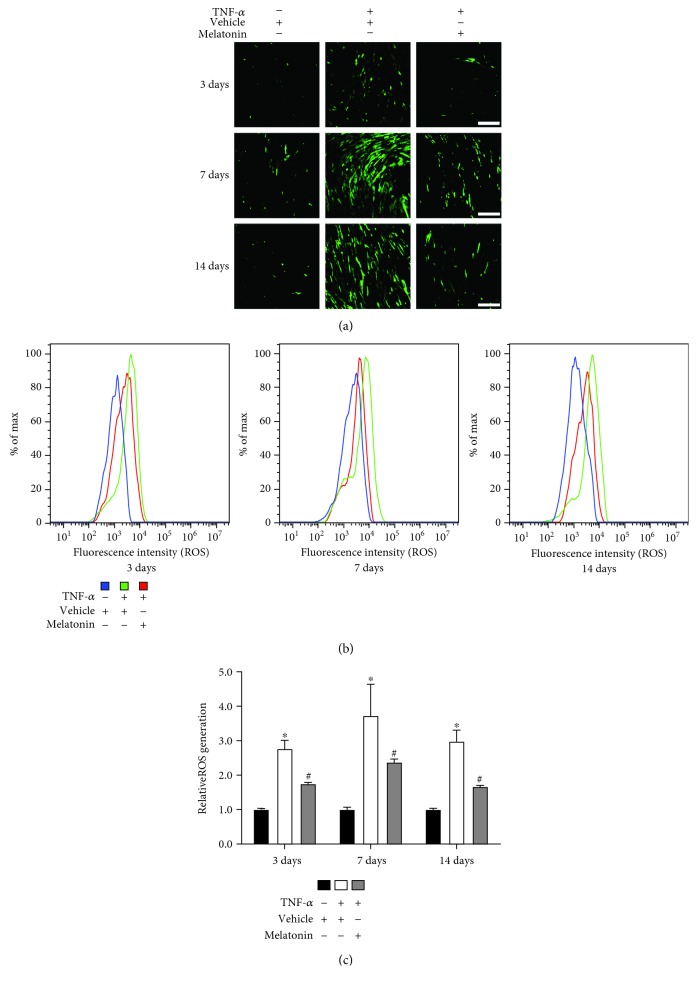
Melatonin decreased TNF-*α*-induced ROS generation during osteogenesis of BMSCs. BMSCs were cultured and underwent osteogenesis in osteogenesis medium containing vehicle (0.05 mol/L ethanol), TNF-*α* (10 ng/mL), or both TNF-*α* (10 ng/mL) and melatonin (100 *μ*mol/L). (a) The adherent cells in a 6-well plate were incubated with DMEM containing CM-H_2_DCFDA (10 *μ*mol/L) for 30 min at 37°C in darkness. Fluorescence was observed by microscopy in a representative sample on days 3, 7, and 14. Green fluorescence indicates ROS-positive cells. Scale bars: 200 *μ*m. (b, c) The cells were resuspended in DMEM containing CM-H_2_DCFDA (10 *μ*mol/L) and incubated for 30 min at 37°C in darkness. Measurement of ROS was analyzed with a flow cytometer. The graphs in (b) represent typical results of fluorescence intensity; the results in (c) are representative of three independent experiments. ^∗^*P* < 0.05 versus the vehicle group, ^#^*P* < 0.05 versus the TNF-*α* group.

To determine whether melatonin can reduce the generation of ROS induced by TNF-*α* during osteogenesis of BMSCs, we induced the human BMSCs to undergo osteogenesis and BMSCs were treated with or without vehicle, TNF-*α* (10 ng/mL), and melatonin (100 *μ*mol/L). As shown in Figure 1(a), TNF-*α* treatment alone increased the ROS fluorescence on days 3, 7, and 14. And melatonin supplementation decreased the ROS fluorescence induced by TNF-*α* during the osteogenic differentiation. Flow cytometry revealed that TNF-*α* treatment rendered the ROS levels higher than those in the vehicle group, whereas melatonin cotreatment decreased the generation of ROS (Figures 1(b) and 1(c)). These results showed that melatonin can reduce the generation of ROS induced by TNF-*α* during osteogenic differentiation.

### 3.2. Melatonin Protected the Osteogenesis of BMSCs in the Presence of TNF-*α*

**Figure 2:**
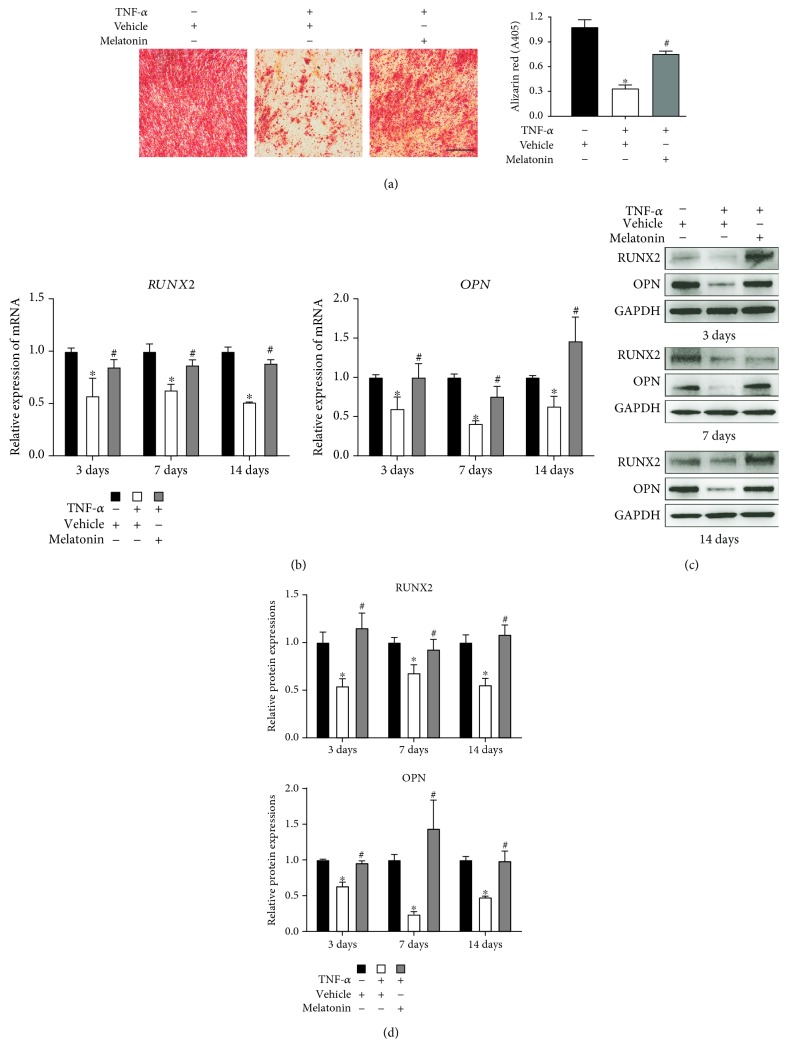
Melatonin protected the osteogenic differentiation of BMSCs in the presence of TNF-*α*. BMSCs were cultured and underwent osteogenesis in osteogenesis medium containing vehicle (0.05 mol/L ethanol), TNF-*α* (10 ng/mL), or both TNF-*α* (10 ng/mL) and melatonin (100 *μ*mol/L). (a) Mineralization of BMSCs after 14 days of osteogenesis was detected by ARS and photographed by microcopy in a representative sample. Scale bars: 200 *μ*m. ARS staining was quantified as the absorbance at 405 nm. (b, c) The expression of runt-related transcription factor-2 (RUNX2) and osteopontin (OPN) was examined using (b) reverse transcription quantitative polymerase chain reaction and (c) Western blot after 3, 7, and 14 days of osteogenic differentiation. (d) The relative protein expressions of RUNX2 and OPN were established. The results are representative of three independent experiments. ^∗^*P* < 0.05 versus the vehicle group, ^#^*P* < 0.05 versus the TNF-*α* group.

To investigate whether melatonin can protect the osteogenesis of BMSCs in the presence of TNF-*α*, we induced osteogenesis in human BMSCs and treated them with or without vehicle, TNF-*α* (10 ng/mL), and melatonin (100 *μ*mol/L). After 14 days of osteogenic differentiation, Alizarin red S (ARS) staining revealed that TNF-*α* supplementation alone reduced the calcium deposits induced by osteoblast and melatonin can counteract their interference mineralization (Figure 2(a)). RT-qPCR and Western blot showed that TNF-*α* treatment downregulated the levels of expression of osteogenic genes, including RUNX2 and OPN, on day 14 after osteogenic differentiation. Similar outcomes were demonstrated on days 3 and 7. After the addition of melatonin to TNF-*α*, the inhibitory effect of TNF-*α* was reversed (Figures 2(b)–2(d)). These findings showed that melatonin can protect the osteogenic differentiation of BMSCs in the presence of TNF-*α*.

### 3.3. Melatonin Inhibited the Expression of Oxidative Enzymes during Osteogenesis of BMSCs

**Figure 3:**
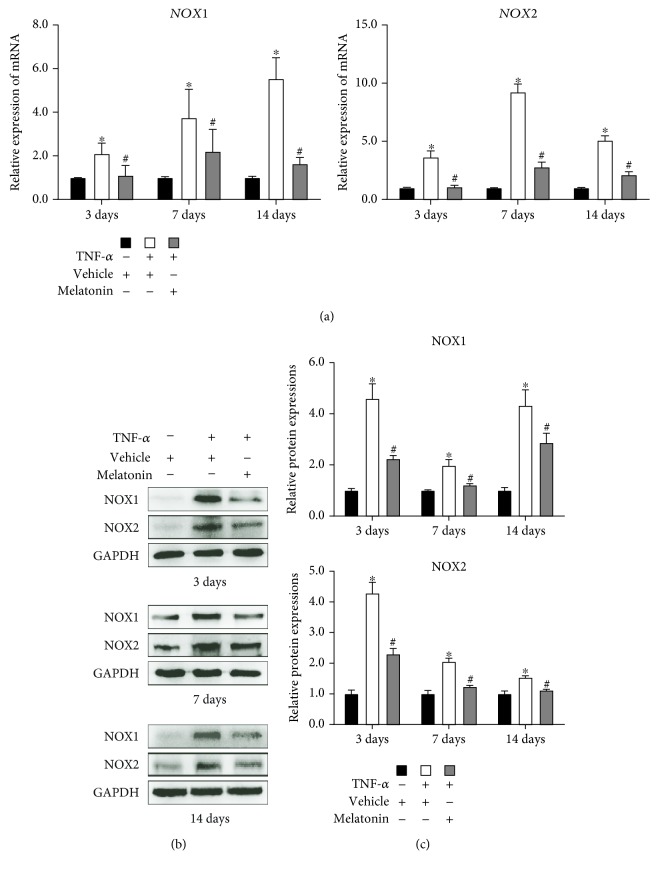
Melatonin inhibited the expression of oxidative enzymes induced by TNF-*α* during the osteogenesis of BMSCs. BMSCs were cultured and underwent osteogenesis in osteogenesis medium containing vehicle (0.05 mol/L ethanol), TNF-*α* (10 ng/mL), or both TNF-*α* (10 ng/mL) and melatonin (100 *μ*mol/L). The expression of NADPH oxidase1 (NOX1) and NADPH oxidase2 (NOX2) was assessed using (a) reverse transcription quantitative polymerase chain reaction and (b) Western blot after 3, 7, and 14 days of osteogenic differentiation. (c) The relative protein expressions of NOX1 and NOX2 were established. The results are representative of three independent experiments. ^∗^*P* < 0.05 versus the vehicle group, ^#^*P* < 0.05 versus the TNF-*α* group.

To determine whether melatonin can inhibit the expression of oxidative enzymes during osteogenic differentiation, we induced the human BMSCs to undergo osteogenesis and BMSCs were treated with or without vehicle, TNF-*α* (10 ng/mL), and melatonin (100 *μ*mol/L). RT-qPCR and Western blot showed the levels of expression of oxidative enzymes, including NOX1 and NOX2, to be enhanced in the presence of TNF-*α*, while the addition of melatonin to TNF-*α* significantly reduced the expression of NOX1 and NOX2 (Figure 3). These results showed that melatonin can inhibit the expression of oxidative enzymes during osteogenic differentiation.

### 3.4. Melatonin Improved the Expression of Antioxidative Enzymes during Osteogenesis of BMSCs

**Figure 4:**
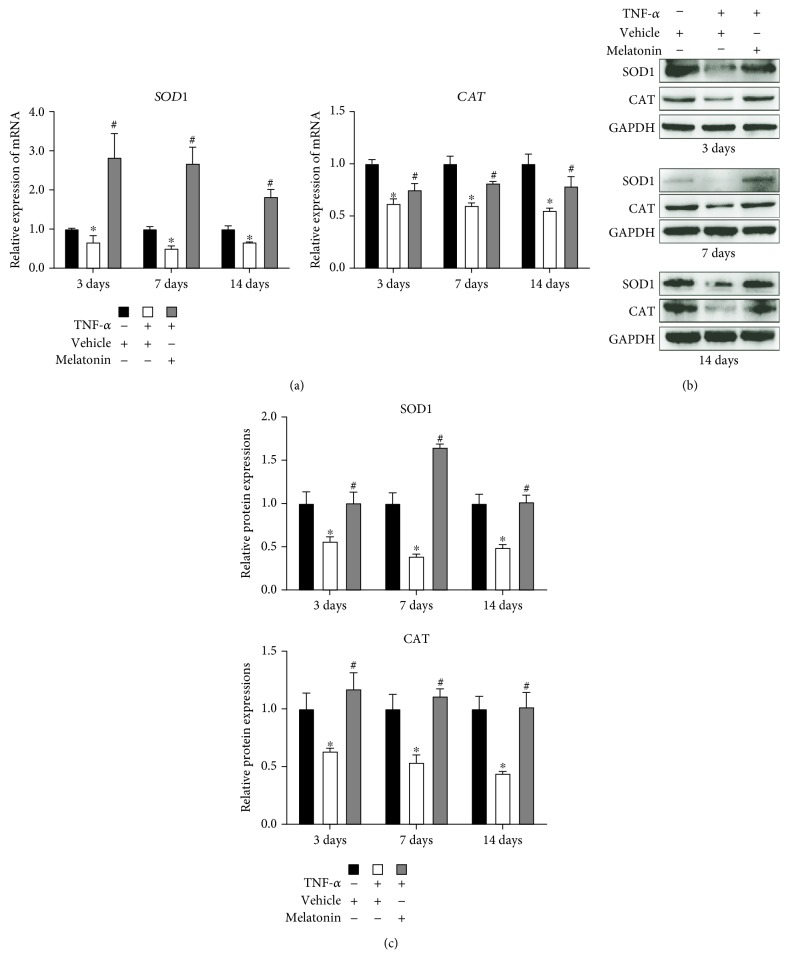
Melatonin improved the expression of antioxidative enzymes during the osteogenic differentiation of BMSCs. BMSCs were cultured and underwent osteogenesis in osteogenesis medium containing vehicle (0.05 mol/L ethanol), TNF-*α* (10 ng/mL), or both TNF-*α* (10 ng/mL) and melatonin (100 *μ*mol/L). The expression of Cu/Zn superoxide dismutase (Cu/Zn SOD or SOD1) and catalase (CAT) was detected using (a) reverse transcription quantitative polymerase chain reaction and (b) Western blot after 3, 7, and 14 days of osteogenic differentiation. (c) The relative protein expressions of SOD1 and SOD2 were established. The results are representative of three independent experiments. ^∗^*P* < 0.05 versus the vehicle group, ^#^*P* < 0.05 versus the TNF-*α* group.

To investigate whether melatonin can improve the expression of antioxidative enzymes during osteogenic differentiation, we induced the human BMSCs to undergo osteogenesis and BMSCs were treated with or without vehicle, TNF-*α* (10 ng/mL), and melatonin (100 *μ*mol/L). RT-qPCR and Western blot indicated that TNF-*α* decreased the level of expression of SOD1 and CAT on days 3, 7, and 14. Melatonin treatment significantly upregulated the expression level of these genes (Figure 4). These results showed that melatonin can improve the expression of antioxidative enzymes during osteogenesis of BMSCs in the presence of TNF-*α*.

### 3.5. Melatonin Inhibited TNF-*α*-Induced NF-*κ*B Signaling Activation during Osteogenesis of BMSCs

**Figure 5:**
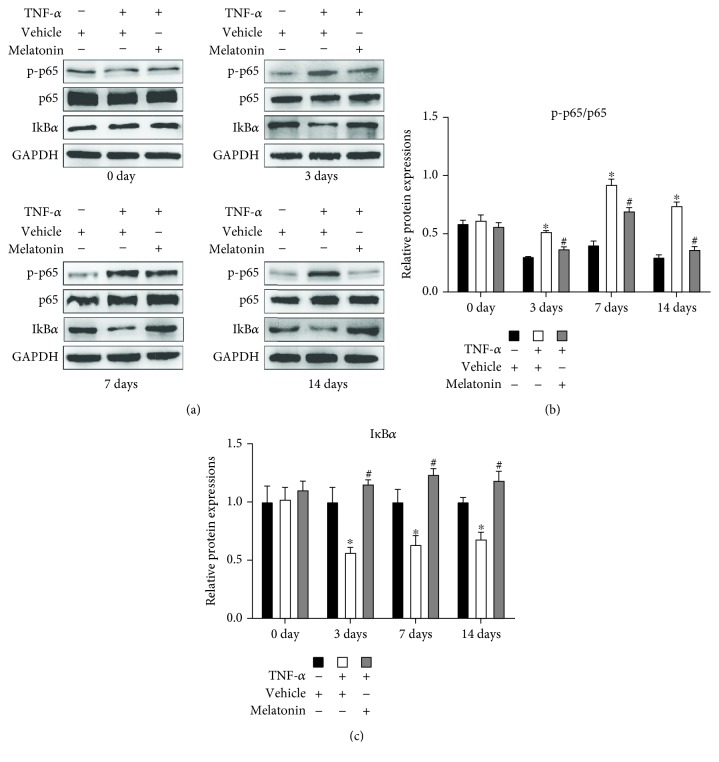
Melatonin suppressed the phosphorylation of NF-*κ*B signaling induced by TNF-*α*. BMSCs were cultured and underwent osteogenesis in osteogenesis medium containing vehicle (0.05 mol/L ethanol), TNF-*α* (10 ng/mL), or both TNF-*α* (10 ng/mL) and melatonin (100 *μ*mol/L). The expression of p65 and I*κ*B*α* protein and phosphor-p65 (p-p65) was detected using (a) Western blot after 0, 3, 7, and 14 days of osteogenic differentiation. (b) Ratio of relative protein expression of p-p65 to relative protein expression of p65 and (c) the relative expression of I*κ*B*α* was examined. The results are representative of three independent experiments. ^∗^*P* < 0.05 versus the vehicle group, ^#^*P* < 0.05 versus the TNF-*α* group.

To investigate the mechanism underlying the antioxidative effect of melatonin in the presence of TNF-*α*, we induced the human BMSCs to undergo osteogenesis and BMSCs were treated with or without vehicle, TNF-*α* (10 ng/mL), and melatonin (100 *μ*mol/L). Western blot showed that TNF-*α* supplementation alone can significantly increase the phosphorylation of p65 protein and the degradation of I*κ*B*α* on days 3, 7, and 14 over levels observed on day 0. The addition of melatonin to TNF-*α* attenuated the effect (Figure 5). These results show that melatonin can inhibit NF-*κ*B signaling pathway activation in the presence of TNF-*α*.

## 4. Discussion

Accumulation of ROS induced by TNF-*α* can significantly inhibit the osteogenic differentiation of BMSCs, which contributes to the bone formation disorders and imbalance of bone remodeling. This process ultimately leads to osteoporosis. Our study investigated that melatonin can decrease the generation of ROS and protect osteogenic differentiation of BMSCs from the inflammatory microenvironment induced by TNF-*α*. That is, these observations described the antioxidative effect of melatonin in the osteogenic differentiation of BMSCs and provide a new theoretical basis for melatonin to maintain the osteogenic ability of BMSCs. Our study demonstrated that melatonin may have a bright future as a candidate drug for the treatment of osteoporosis.

In 1956, Harman [22] first proposed the oxidative stress hypothesis that the cellular damage caused by ROS is the main cause of aging. ROS are also involved in the pathological processes of various aging-related diseases, such as chronic obstructive pulmonary disease, diabetes, atherosclerosis, and Alzheimer's disease [23, 24]. In the skeletal system, ROS are closely related to the occurrence and development of osteoporosis [25]. One of the main reasons is that ROS can significantly inhibit osteogenic differentiation of BMSCs. It has been reported that ROS generation impairs several osteogenesis-related signaling pathways, such as Wnt/*β*-catenin signaling and Hedgehog signaling [1]. Almeida et al. [26] also demonstrated that increased oxidant stress could bring on reduction of osteoblasts and impairment of bone formation in vivo. In other words, the proliferation of ROS has a huge inhibitory effect on osteogenesis of BMSCs and bone formation.

The formation of the inflammatory microenvironment in the bone marrow accelerates the production and accumulation of ROS and so exacerbates the impaired osteogenesis. It has been reported that TNF-*α* strongly inhibits the bone formation both by reducing osteogenesis and by improving osteoclastogenesis [27], in which several signaling pathways are involved. Furthermore, TNF-*α* can increase the expression of NOX by activating NF-*κ*B signaling, thus inducing the generation of ROS [4]. Based on the relevant literature and our previous study, we used the concentration of 10 ng/mL TNF-*α* to simulate the inflammation microenvironment, induce ROS production, and explore the relationship between ROS and osteogenic differentiation of BMSCs in this study. We found that after the addition of TNF-*α*, the ROS generation markedly increased (Figure 1). The TNF-*α* supplementation suppressed the expression of osteogenic markers (RUNX2 and OPN) and inhibited the osteogenic differentiation of BMSCs (Figure 2), suggesting that by overcoming the excessive amount of ROS, it might be a positive way to counteract the inhibition of osteogenesis.

The generation and elimination of ROS depend mainly on the balance between oxidase and antioxidase. NADPH oxidative enzymes, such as NOX1 and NOX2, are among the major sources of ROS. The presence of inflammatory cytokines upregulates the expression of NADPH oxidative enzymes in bone and joint disease and consequently enhances the proliferation of ROS [28]. In our study, we found that the expression of NOX1 and NOX2 was remarkably increased in the presence of TNF-*α* (Figure 3). Moreover, the antioxidant enzymes (i.e., SOD1 and CAT) were detected in our study. Superoxide dismutase (SOD) is an enzyme that can catalyze the dismutation of O_2_^−^ radical into either oxygen or H_2_O_2_, and catalase (CAT) can catalyze H_2_O_2_ into oxygen and water. TNF-*α* treatment can reduce the expression of SOD1 and CAT, which indicated that there is an imbalance between the oxidase system and the antioxidase system during ROS production in the presence of TNF-*α*.

Melatonin has been recognized as an antioxidative molecule. In addition to directly scavenging oxygen free radicals, melatonin can also upregulate the expression of antioxidase and downregulate the expression of oxidase. It has been reported that melatonin can be classified as a mitochondria-targeted antioxidant [12]. Recently, several studies have been reported that melatonin can act as an antioxidant and participate in the survival and differentiation of MSCs. Zhu et al. [29] reported that melatonin can protect adipose-derived MSCs from ROS and promote their therapeutic potency in a rat model. Liu et al. [30] demonstrated that melatonin, by suppressing ROS, can counteract the inflammatory effects of cytokines, which would otherwise inhibit the chondrogenesis of synovial membrane MSCs. However, whether melatonin can play a similar role during osteogenic differentiation of MSCs in the inflammatory environment especially in the presence of TNF-*α* requires further confirmation. According to previous studies [17, 19], when the concentration of melatonin is 100 *μ*mol/L, the proosteogenic, anti-inflammatory, and antioxidative effects of melatonin can be maximized. In the present study, we induced BMSCs, which have a stronger osteogenesis potential than other sources of MSCs, to undergo osteogenic differentiation, and we found that melatonin can decrease the generation of ROS—which had been significantly increased by TNF-*α* supplementation—to a remarkable degree (Figure 1), through downregulating the expression of NOX1 and NOX2 (Figure 3) and upregulating the expression of SOD1 and CAT (Figure 4). Then, we found that melatonin can significantly reverse the inhibition of osteogenesis (Figure 2), as had also been demonstrated in our previous study [19]. Our findings further supported the viewpoint that melatonin has an antioxidative effect, in accordance with another study that found that melatonin protects osteogenesis of MSCs in the presence of another inflammatory cytokine, interleukin 1 beta (IL-1*β*) [31].

The NF-*κ*B signaling pathway is recognized as the main downstream target of TNF-*α*. A growing evidence has revealed that the activation of NF-*κ*B signaling contributes to the TNF-*α*-inhibited osteogenesis of MSCs [32, 33]. Moe et al. [34] reported that TNF-*α* can activate the NF-*κ*B signaling and upregulate the expression of NADPH oxidases, which contribute to a high level of ROS. In this study, we examined the expression and phosphorylation level of the NF-*κ*B pathway protein at different points in time during BMSC osteogenic differentiation. Results showed that the NF-*κ*B pathway is continuously activated after TNF-*α* treatment (Figure 5), contributing to the continuous high expression of NOX and high level of ROS. TNF-*α*-induced ROS generation can subsequently cause the continued activation of NF-*κ*B signaling and promote TNF-*α* expression, constituting a positive feedback loop and forming a vicious cycle [35]. After melatonin cotreatment, the NF-*κ*B signaling activity was blocked (Figure 5). We conjectured that the continuous activation of NF-*κ*B signaling might be relative to the crosstalk between TNF-*α* and ROS and that melatonin might break this cycle. Accordingly, we concluded that melatonin may exert its antioxidative effects at least partially through inactivation of the NF-*κ*B pathway.

Our study had some limitations. First, we used only one of the inflammatory cytokines, TNF-*α*, to induce ROS production and impair osteogenic differentiation of BMSCs. This unilaterally demonstrated that melatonin can reverse the inhibition of osteogenic differentiation of BMSCs caused by TNF*α*-induced ROS. Whether melatonin can antagonize the ROS induced by other inflammatory cytokines, such as IL-1 and IL-6, still needs further investigation. Second, our research on underlying mechanisms was not sufficiently complete. We found that after TNF-*α* treatment, the NF-*κ*B pathway was activated, with increased I*κ*B*α* degradation and p65 phosphorylation. Melatonin supplementation could significantly reduce this. However, the role and underlying mechanisms of melatonin on specifically inhibiting the activation of NF-*κ*B signaling induced by TNF-*α* also need further study. At last, our study was in vitro research only. Future studies should focus on the experiments in an animal model that could provide in vivo evidence to verify the antioxidative role of melatonin on osteogenesis of BMSCs.

## 5. Conclusions

In conclusion, the present study showed that the generation of ROS, which can be induced by TNF-*α*, is involved in the inhibition of osteogenesis differentiation of BMSCs; melatonin can protect against the oxidant damage by suppressing oxidant enzymes and improving antioxidant enzymes. This process may be associated with the activation and inactivation of the NF-*κ*B signaling pathway. Our previous studies had already showed that melatonin plays a vital role in promoting osteogenesis of BMSCs. For this reason, we consider that through its antioxidative, anti-inflammatory, and proosteogenic effects, melatonin may have a bright future as a candidate drug for maintaining the osteogenic ability of BMSCs and treating osteoporosis.

## Figures and Tables

**Table 1:** All primers for reverse transcription quantitative polymerase chain reaction.

Gene	Primer sequence (5′-3′)
*GAPDH*	Forward: AGAAAAACCTGCCAAATATGATGAC
	Reverse: TGGGTGTCGCTGTTGAAGTC

*RUNX2*	Forward: AGAAGGCACAGACAGAAGCTTGA
	Reverse: AGGAATGCGCCCTAAATCACT

*OPN*	Forward: GCGAGGAGTTGAATGGTG
	Reverse: CTTGTGCTGTGGGTTTC

*NOX1*	Forward: GCACACCTGTTTAACTTTGACTG
	Reverse: GGACTGGATGGGATTTAGCCA

*NOX2*	Forward: AACGAATTGTACGTGGGCAGA
	Reverse: GAGGGTTTCCAGCAAACTGAG

*SOD1*	Forward: GGTGGGCCAAAGGATGAAGAG
	Reverse: CCACAAGCCAAACGACTTCC

*CAT*	Forward: TGGGATCTCGTTGGAAATAACAC
	Reverse: TCAGGACGTAGGCTCCAGAAG

## Data Availability

The datasets analyzed during the current study are available from the corresponding authors on reasonable request.
